# The effects of redox controls mediated by glutathione peroxidases on root architecture in *Arabidopsis thaliana*

**DOI:** 10.1093/jxb/ert486

**Published:** 2014-01-27

**Authors:** Gisele Passaia, Guillaume Queval, Juan Bai, Marcia Margis-Pinheiro, Christine H. Foyer

**Affiliations:** ^1^Centre for Plant Sciences, School of Biology, Faculty of Biological Sciences, University of Leeds, Leeds LS2 9JT, UK; ^2^Depto. Genética, Universidade Federal do Rio Grande do Sul, Av. Bento Gonçalves 9500, Prédio 43.312, CEP 91501–970 Porto Alegre, RS, Brazil; ^3^College of Life Science, Northwest A&F University, Shaanxi 712100, China

**Keywords:** Abscisic acid, auxin, glutathione, oxidative stress, redox regulation, root architecture, strigolactones.

## Abstract

Glutathione peroxidases (GPXs) fulfil important functions in oxidative signalling and protect against the adverse effects of excessive oxidation. However, there has been no systematic characterization of the functions of the different GPX isoforms in plants. The roles of the different members of the *Arabidopsis thaliana* GPX gene (*AtGPX*) family were therefore investigated using *gpx1*, *gpx2*, *gpx3*, *gpx4*, *gpx6*, *gpx7*, and *gpx8* T-DNA insertion mutant lines. The shoot phenotypes were largely similar in all genotypes, with small differences from the wild type observed only in the *gpx2*, *gpx3*, *gpx7*, and *gpx8* mutants. In contrast, all the mutants showed altered root phenotypes compared with the wild type. The *gpx1*, *gpx4*, *gpx6*, *gpx7*, and *gpx8* mutants had a significantly greater lateral root density (LRD) than the wild type. Conversely, the *gpx2* and *gpx3* mutants had significantly lower LRD values than the wild type. Auxin increased the LRD in all genotypes, but the effect of auxin was significantly greater in the *gpx1*, *gpx4*, and *gpx7* mutants than in the wild type. The application of auxin increased *GPX4* and *GPX7* transcripts, but not *GPX1* mRNAs in the roots of wild-type plants. The synthetic strigolactone GR24 and abscisic acid (ABA) decreased LRD to a similar extent in all genotypes, except *gpx6*, which showed increased sensitivity to ABA. These data not only demonstrate the importance of redox controls mediated by AtGPXs in the control of root architecture but they also show that the plastid-localized *GPX1* and *GPX7* isoforms are required for the hormone-mediated control of lateral root development.

## Summary

We demonstrate that the GPX proteins are important in the control of root architecture and that loss of any of the GPX isoforms exerts an influence on lateral root density.

## Introduction

Plant cells contain a large array of antioxidant enzymes that control the metabolism of oxidants such as reactive oxygen species (ROS) and also play a role in redox signalling (Foyer and Noctor, 2009, 2011). The induction of antioxidant enzymes is a particularly important component of plant stress responses that limit oxidant-induced programmed cell death (PCD) (Wu *et al.*, 2010). Within the antioxidant network of plant cells, ascorbate peroxidases reduce H_2_O_2_ at the expense of ascorbate, which is then regenerated by monodehydroascorbate and dehydroascorbate reductases (MDHAR and DHAR). In parallel, glutathione peroxidases (GPXs), glutathione *S*-transferases (GSTs), and peroxiredoxins (PRXs) reduce H_2_O_2_ and hydroperoxides by ascorbate-independent thiol-mediated pathways (Dietz *et al.*, 2002; Chang *et al.* 2009). While many classes of GSTs display peroxidase activity (Dixon *et al.*, 2009), in general they are only able to metabolize H_2_O_2_ at low rates (Mannervik, 1985).

GPXs are non-haem thiol hydroperoxidases, which catalyse the reduction of H_2_O_2_ or organic hydroperoxides to water or corresponding alcohols (Herbette *et al.*, 2007). The active site of these enzymes is composed of a catalytic triad formed by selenocysteine/cysteine, glutamine, and tryptophan (Herbette *et al.*, 2007). In contrast to the animal GPXs, which are selenoproteins containing a selenocysteine at the catalytic site, the plant enzymes do not bind selenium (Eshdat *et al.*, 1997; Herbette *et al.*, 2007; Toppo *et al.*, 2008). Moreover, the animal GPXs exclusively use GSH as the reducing substrate but, despite their current nomenclature, the plant GPXs prefer reduced thioredoxin (TRX) as reductant and they have comparatively low activities with GSH (Iqbal *et al.*, 2006; Navrot *et al.*, 2006; Herbette *et al.*, 2007). They are therefore better described as thiol peroxidases (Navrot *et al.*, 2006).

GPXs are considered to play important roles in preventing oxidant-induced PCD. In *Chlamydomonas reinhardtii*, for example, a *GLUTATHIONE PEROXIDASE HOMOLOGOUS* (*GPXH*) gene is highly expressed in cells exposed to high light or singlet oxygen generation in the chloroplasts (Leisinger *et al.*, 2001; Fischer *et al.*, 2007; Ledford *et al.*, 2007). Constitutive over-expression of *GPXH* together with *GSTS1* enhanced tolerance to singlet oxygen, suggesting that *GPXH* is important in defences against photooxidative stress.

GPXs interact with other proteins and hence they are considered to have signalling functions (Delaunay *et al.*, 2002; Miao *et al.*, 2006). Some members of the GPX superfamily can form tetrameric structures with other proteins in response to oxidants such as H_2_O_2_ or hydroperoxides. In yeast, for example, GPX3 is considered to activate the Yeast Activation Protein 1 (Yap-1) transcription factor via an oxidation-induced event in order to promote the activation of genes encoding thiol-reducing and antioxidant proteins (Delaunay *et al.*, 2002). The *Arabidopsis* AtGPX3 interacts with protein phosphatase type 2C (PP2C) proteins such as ABSCISIC ACID INSENSITIVE (ABI) 1 and ABI2, in order to activate plasma membrane Ca^2+^ and K^+^ channels that facilitate stomatal closure (Miao *et al.*, 2006). Rice plants lacking GPX3 had shorter roots and smaller shoots than wild-type plants, indicating that GPX3 plays an essential role in root and shoot development (Passaia *et al.*, 2013). Two stromal GPXs (AtGPX1 and AtGPX7) were shown to be important in chloroplast functions, particularly light acclimation and also in plant immune responses (Chang *et al.*, 2009).

In *Arabidopsis thaliana*, GPXs are encoded by a small gene family, named *AtGPX1* to *AtGPX8*. The enzymes products of these genes are considered to be important redox sensors as well as antioxidants, but the precise functions of the different GPX isoforms remains poorly characterized. Each GPX member localizes to a distinct cellular location. While there is some controversy in the literature regarding the intracellular localizations of AtGPX3 and AtGPX6, there is a consensus that AtGPX1 and AtGPX7 are localized to the chloroplasts, AtGPX4 and AtGPX5 are cytosolic, AtGPX2 is localized to the endoplasmic reticulum/cytosol, and AtGPX8 to the cytosol/apoplast (Rodriguez Milla *et al.*, 2003; Margis *et al.*, 2008) or the cytosol and nucleus (AtGPX8; Gaber *et al.*, 2012). Phylogenetic analysis indicates that the AtGPX3 protein is localized to the endoplasmic reticulum/cytosol and that AtGPX6 is localized in the mitochondria (Margis *et al.*, 2008). Each isoform within a given cellular compartment is considered to fulfil distinct functions. For example, AtGPX8 protects the nucleus and cytosol from the harmful effects of uncontrolled oxidation (Gaber *et al.*, 2012). However, there has been no systematic characterization of the functions of the different GPX isoforms in *Arabidopsis.* Moreover, the precise roles of each GPX isoform in leaves and roots remain largely uncharacterized. The following experiments were therefore undertaken to explore the roles of the different AtGPX isoforms using *gpx1*, *gpx2*, *gpx3*, *gpx4*, *gpx6*, *gpx7*, and *gpx8* T-DNA insertion mutant lines. A preliminary characterization of the different mutant lines revealed that the shoot phenotypes were largely similar in all genotypes, regardless of the GPX composition. Due to the absence of differences in shoot parameters between the *gpx* mutants and the wild type, the focus of this study was on the role of the different GPX isoforms in the control of root architecture, where significant differences were observed between the *gpx* mutants and the wild type. The aim of this study was to characterize the root phenotypes observed in the *gpx* mutants in relation to the action of hormones that control root architecture, particularly auxin, strigolactones (SLs), and abscisic acid (ABA).

## Materials and methods

### Identification and isolation of T-DNA insertion mutants

T-DNA insertion lines from SAIL (Sessions *et al.*, 2002), SALK (Alonso *et al.*, 2003), and WiscDsLox (Woody *et al.*, 2007) collections were obtained. Donor stock numbers SALK_027373 (*gpx1*; At2g25080), SALK_082445 (*gpx2*; At2g31570), SAIL_278_E06 (*gpx3-1*; At2g43350), SALK_071176 (*gpx3-2*; At2g43350), SALK_139870 (*gpx4*; At2g48150), WISCDSLOX_321_H10 (*gpx6*; At4g11600), SALK_023283 (*gpx7*; At4g31870), and SALK_127691 (*gpx8*; At1g63460) were obtained from the ABRC (Ohio State University) and NASC (Nottingham Arabidopsis Stock Center). All T-DNA mutants except *gpx3-1* are in the Col-0 background; *gpx3-1* is in the Col-3 background. Plants were germinated and grown in conventional soil and, to confirm the position and the homozygosity of the insertion, PCR was performed with genomic DNA using gene-specific oligonucleotides whose sequences are given in Supplementary Table S2 available at *JXB* online. The position of the T-DNA insertion sequence and oligonucleotides for each genotype are shown in Supplementary Fig. S1 available at *JXB* online.

Upon request, all novel materials described in this publication will be made available in a timely manner for non-commercial research purposes, subject to the requisite permission from any third-party owners of all or parts of the material.

### Growth on soil and rosette measurements

Wild types (Col-0 and Col-3) and *gpx1*, *gpx2*, *gpx3-1*, *gpx6*, *gpx7*, and *gpx8* mutant seeds were grown on compost in controlled environment chambers under optimal growth conditions under either short (8h) photoperiod or long (16h) photoperiod conditions for 4 weeks. Rosette diameters were measured every 3 d. Leaf number, leaf fresh and dry weight measurements, and leaf pigment determinations were made on material harvested at 27/28 d.

### Growth on plates and hormone treatments

Seeds of the wild type (Col-0) and *gpx1*, *gpx2*, *gpx3-2*, *gpx4*, *gpx6*, *gpx7*, and *gpx8* mutants were surface sterilized for 2min in 75% ethanol and 5min in 4% sodium hypochlorite, and washed several times in sterilized water until the pH was ~7. Sterilized seeds were then sown on 12cm square plates containing half-strength Murashige and Skoog medium (1/2 MS medium, pH 5.7) with 0.01% myo-inositol, 0.05% MES, 1% sucrose, and 0.8% plant agar. Plates were stored for 2 d in the cold and dark to synchronize germination and were placed vertically in a plant growth cabinet with a 16h photoperiod and a temperature of 22 °C. After 3 d, the seedlings were gently transferred using forceps to new plates containing the same medium plus 2 μM GR24 (synthetic SL) and grown for a further 5 d. Auxin treatments were performed by transferring the seedling after a 5 d growth period to 1/2 MS medium containing 1 μM NAA (1-naphthaleneacetic acid) for 3 d. ABA was added to the media at concentrations of 0.3 μM or 0.5 μM. Seeds were placed directly on ABA-containing medium, cold treated, and grown as above for 8 d.

### Root architecture

The root length and number of lateral roots formed were analysed on 8-day-old seedlings. Photos were taken and the root length was measured using ImageJ software. Lateral root density (LRD) was calculated by dividing the number of visible lateral roots by the primary root length for each root analysed.

### Root staging

Measurements of the stages of lateral root development were performed as described previously (Malamy and Benfey, 1997). Roots were incubated in 0.24 N HCl and 20% methanol at 62 °C for 20min. This solution was then replaced with 7% NaOH with 60% ethanol and the roots were then incubated for a further 15min at room temperature. Following rehydration in 40% ethanol, 20% ethanol, and 10% ethanol, the roots were infiltrated for 15min in 5% ethanol with 25% glycerol. The root samples were then maintained in 50% glycerol until microscopic analysis (M 4000-D, Swift). The stages of primordium development were classified as described by Péret *et al.* (2009) as follows: stage I (single layered primordium composed of up to 10 small cells of equal length formed from individual or a pair of pericycle founder cells), stage II (periclinal cell division forming an inner and an outer layer), stages III, IV, V, VI, and VII (anticlinal and periclinal divisions create a dome-shaped primordium), and stage VIII (emergence of the primordium from the parental root).

### Gene expression analysis

RNA was extracted from the shoots and roots of 10-day-old Col-0, *gpx1*, *gpx2*, *gpx3-2*, *gpx4*, *gpx6*, *gpx7*, and *gpx8* plantlets grown in 1/2 MS medium in a growth chamber at 20 °C with a 16h photoperiod using an RNeasy Plant Mini Kit (Qiagen, Hilden, Germany). For each genotype, three biological replicates for the shoot and the root were prepared. Reverse transcription of 1 μg of RNA into cDNA was performed using the QuantiTect Reverse Transcription Kit (Qiagen). The qPCR was performed on 20ng of cDNA with a QuantiFast SYBR Green PCR kit (Qiagen) in the presence of 0.5 μM primers in a CFX96 thermocycler (Biorad, Hercules, CA, USA). PCR conditions were as follows: 5min 95 °C, 45 cycles 10 s 95°C and 30 s 60°C. Additionally melting curve analysis was performed at the end of each run to ensure specificity of the products. The mean value of three replicates was normalized using *Actin2* as internal control (primer sequences can be found in Supplementary Table S2 available at *JXB* online). The quality of amplification curves was checked with the LinRegPCR software (Ramakers *et al.*, 2003) and curves with R >0.995 and efficiencies between 1.9 and 2.1 were kept for the following expression value calculations. All expression data analyses were performed after comparative quantification of amplified products by using the 2^–ΔΔCt^ method (Livak and Schmittgen, 2001; Schmittgen and Livak, 2008).

### Statistical analysis

Data represent the mean ±standard error of the mean (SEM). Statistical analysis was performed by Student’s *t*-test. The values were considered statically different when *P* was <0.05.

## Results

### Relative abundance of *AtGPX* mRNAs in the *gpx1*, *gpx2*, *gpx3*, *gpx4*, *gpx6*, *gpx7*, and *gpx8* mutants compared with the wild type

**Fig. 1.**
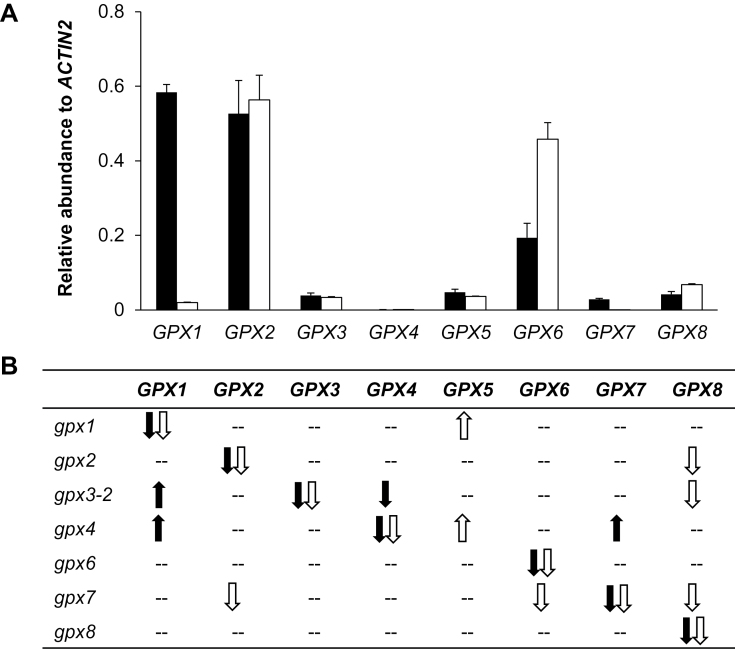
The relative expression of *GPX* genes in Col-0 (A) and in the different *gpx* mutants relative to the wild type (B). Black bars and arrows, shoots; white bars and arrows, roots. Data are the mean ±SE of 3–6 biological replicates.

As a first step in the characterization of the functions of the different AtGPX isoforms, the relative abundance of the different *AtGPX* transcripts was compared in the roots and shoots of the *gpx1*, *gpx2*, *gpx3-2*, *gpx4*, *gpx6*, *gpx7*, and *gpx8* mutants relative to the wild type (Col-0; Fig. 1; Supplementary Table S1 available at *JXB* online). *AtGPX1* and *AtGPX2* transcripts were abundant in shoots. The level of *AtGPX2* transcripts was highest in roots, as was the level of *AtGPX6* mRNAs (Fig. 1A). *AtGPX4* mRNAs were below the level of detection in roots and shoots of the wild-type plants (Fig. 1A). *AtGPX1* mRNAs were more abundant in the shoots of *gpx3-2* and *gpx4* mutants. The *AtGPX2* transcripts were less abundant in the roots of the *gpx7* mutants. *AtGPX4* transcripts were less abundant only in the shoots of the *gpx3-2* mutant. *AtGPX5* mRNA levels were higher in the roots of the *gpx1* and *gpx4* mutants. *AtGPX6* mRNA levels were lower in the root of the *gpx7* mutant. *AtGPX7* transcripts were increased in the shoots of the *gpx4* mutants. *AtGPX8* transcripts were less abundant in roots of *gpx2*, *gpx3-2*, and *gpx7* mutants relative to the wild type. Gene expression data for each mutant are provided in Supplementary Table S1 available at *JXB* online.

### Shoot phenotypes in the *gpx1*, *gpx2*, *gpx3*, *gpx6*, *gpx7*, and *gpx8* mutants

**Fig. 2.**
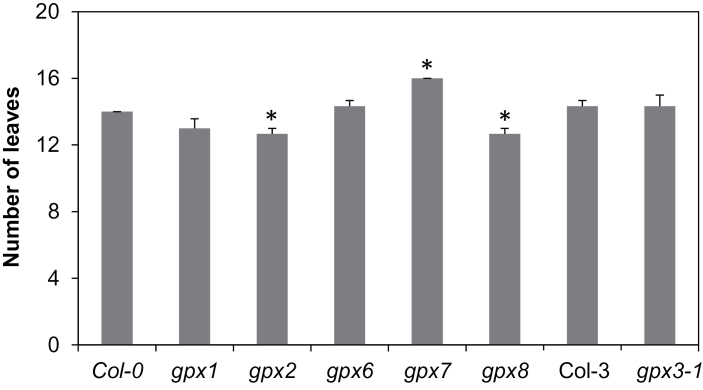
The relative number of leaves in 4-week-old *gpx* mutant rosettes relative to the wild types grown under long day conditions. Data are the mean ±SE of three plants. Asterisks indicate significant differences compared with the wild type, *P*<0.05.

**Fig. 3.**
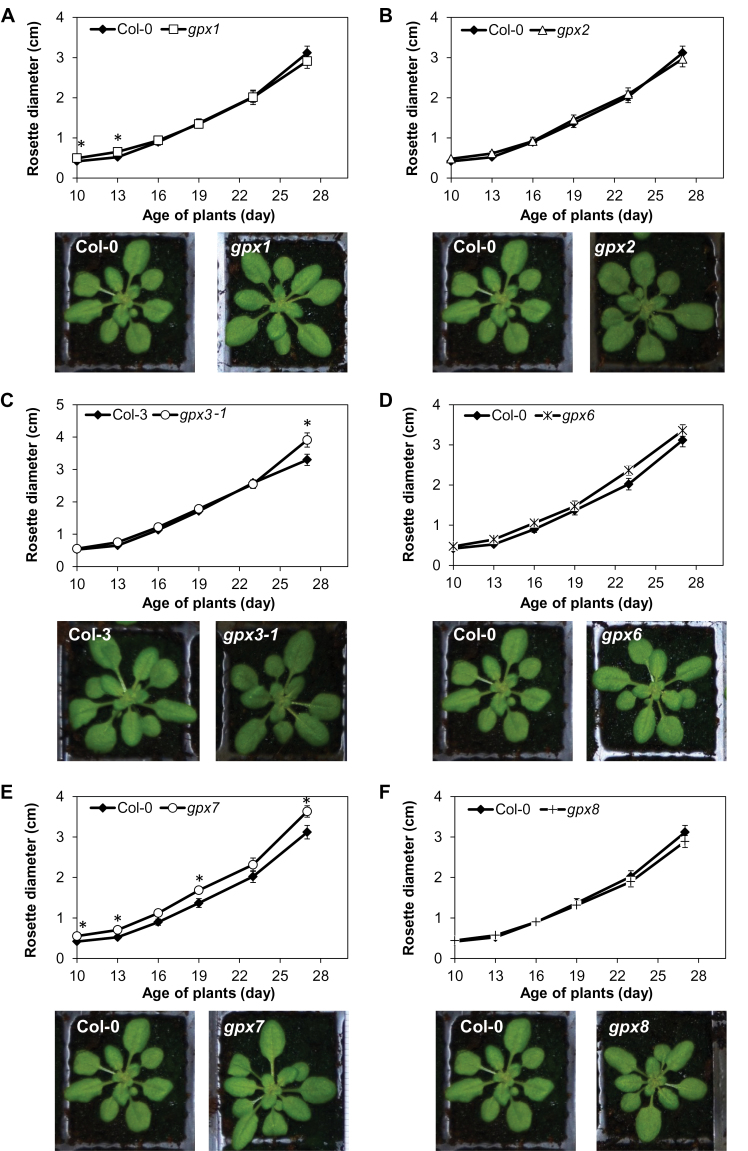
A comparison of rosette phenotypes in 4-week-old *gpx* mutants relative to the wild types grown under short day conditions. (A) *gpx1* mutant; (B) *gpx2* mutant; (C) *gpx3-1* mutant; (D) *gpx6* mutant; (E) *gpx7* mutant; (F) *gpx8* mutant and the respective wild types. Data are the mean ±SE (*n*=10). Asterisks indicate significant differences, *P*<0.05.

In order to determine the importance of the different AtGPX isoforms for shoot parameters, the shoot phenotypes of the different mutants (*gpx1*, *gpx2*, *gpx3-1*, *gpx6*, *gpx7*, and *gpx8*) were compared with those of the wild type after 4 weeks growth under long (16h) photoperiod growth conditions. Shoot biomass (fresh weight per plant) and leaf pigment (chlorophylls and carotene, expressed on a leaf area basis) contents were similar in all the lines (data not shown). However, the *gpx2* and *gpx8* mutants had significantly fewer leaves than the wild-type plants at the 28 d growth stage. In contrast, the *gpx7* mutants had significantly more leaves than the wild type at this point (Fig. 2). To characterize the roles of the different AtGPX isoforms in shoot development further, shoot growth was also examined under short photoperiod (8h) growth conditions. The shoot phenotypes of the mutants were very similar to those of their respective wild types when plants were grown under short day conditions (Fig. 3). However, *gpx3-1* and *gpx7* had a significantly greater rosette diameter than the wild type after 4 weeks under these conditions (Fig. 3C, E).

### Root phenotypes in the *gpx1*, *gpx2*, *gpx3*, *gpx4*, *gpx6*, *gpx7*, and *gpx8* mutants

**Fig. 4.**
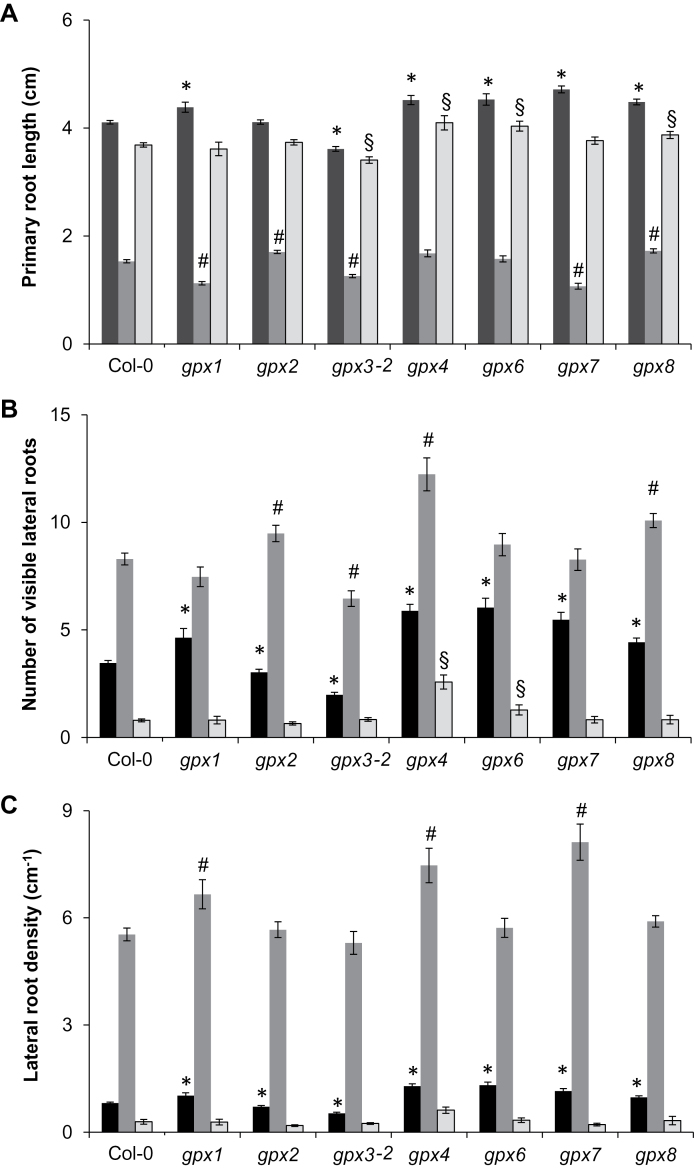
A comparison of the effects of auxin and strigolactone on the root phenotypes in the different *gpx* mutants and the wild type. The primary root length (A), number of visible lateral roots (B), and lateral root density (C) were measured in seedlings of the different *gpx* mutant lines and the wild type (Col-0) grown in either the absence (black bars) or presence of auxin (1 μM NAA; grey bars) or strigolactone (2 μM GR24, light grey bars). Data are the mean ±SE. Symbols indicate significant differences, *P*<0.05 in control conditions (*); after auxin treatment (#); and after strigolactone treatment (§) in the mutants compared with the wild type.

To explore the roles of the different AtGPX isoforms in the control of root development, comparisons of the growth of the primary roots, the number of lateral roots, and LRDs were performed on 8-day-old seedlings (Fig. 4). Root phenotypes were compared in the different mutants (*gpx1*, *gpx2*, *gpx3-1*, *gpx3-2*, *gpx4*, *gpx6*, *gpx7*, and *gpx8*) and the wild types (Col-0 and Col-3) in control conditions and in the presence of hormones. All the mutant lines showed large differences in LRD compared with the wild type in control conditions, with the *gpx1*, *gpx4*, *gpx6*, *gpx7*, and *gpx8* mutants having greater LRD values relative to the wild type (Fig. 4C). In contrast, *gpx2* and *gpx3-2* had a lower LRD than Col-0 (Fig. 4C).

### Auxin-dependent increases in lateral root density

In order to determine whether any of the different AtGPX isoforms were required for the auxin-dependent control of lateral root development, root phenotypes were compared in *gpx1*, *gpx2*, *gpx3-2*, *gpx4, gpx6*, *gpx7*, and *gpx8* and Col-0 seedlings grown either in the absence of added hormone or in the presence of auxin (1 μM NAA; Fig 4). Auxin treatment resulted in a large increase in LRD in all of the genotypes. However, in contrast to the *gpx2* and *gpx3-2* mutants, which showed a response to auxin similar to that of the wild type (Fig. 4A–C), the effect of auxin was significantly greater in the *gpx1*, *gpx4*, and *gpx7* seedlings than in the wild type.

### Strigolactone (GR24)-dependent inhibition of lateral root development

The roles of the AtGPX isoforms in the SL-dependent control of lateral root development were investigated in the *gpx1*, *gpx2*, *gpx3-2*, *gpx4*, *gpx6*, *gpx7*, and *gpx8* mutants. Col-0 and mutant seedlings were grown either in the absence of added hormone or in the presence of the synthetic SL, GR24 (2 μM; Fig. 4). As predicted from the known action of SLs on root architecture, the addition of GR24 inhibited lateral root formation in all the genotypes studied here. The addition of GR24 decreased LRD values to a similar extent in all the genotypes except for the *gpx4* seedlings, which showed a significantly lower response to GR24-induced inhibition of LRD than the wild type (Fig. 4C).

### Abscisic acid-induced inhibition of lateral root development

**Fig. 5.**
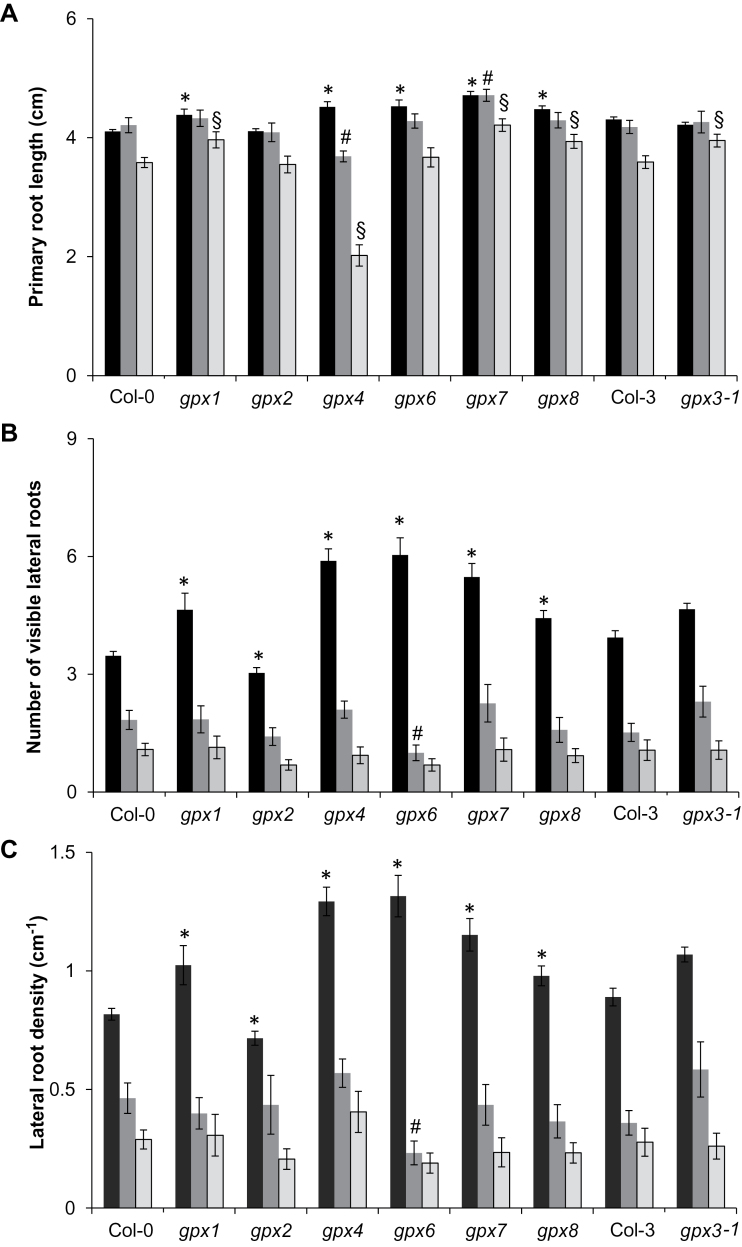
A comparison of the effects of different concentrations of abscisic acid (ABA) on the root phenotypes in the different *gpx* genotypes and their respective wild types. The primary root length (A), number of visible lateral roots (B), and lateral root density (C) were measured in seedlings of the different *gpx* mutant lines and the respective wild types (Col-0 and Col-3) grown in either the absence (black columns) or the presence of 0.3 μM ABA (grey columns) or 0.5 μM ABA (light grey columns). Data are the average ±SE. Symbols indicate significant differences, *P*<0.05 in control conditions (*); after 0.3 μM (#); and 0.5 μM (§) ABA treatments between the wild types and the mutants.

In order to determine whether any of the AtGPX isoforms were required for the ABA-dependent control of lateral root development, root phenotypes were compared in *gpx1*, *gpx2*, *gpx3-1*, *gpx4*, *gpx6*, *gpx7*, *gpx8*, Col-0, and Col-3 seedlings grown either in the absence of added hormone or in the presence of ABA (Fig. 5A–C). Growth in the presence of ABA decreased LRD in all the genotypes; *gpx6* showed enhanced sensitivity to ABA-induced inhibition of lateral root development (Fig. 5).

### Effects of auxin on the developmental stages of the lateral root primordia

**Fig. 6.**
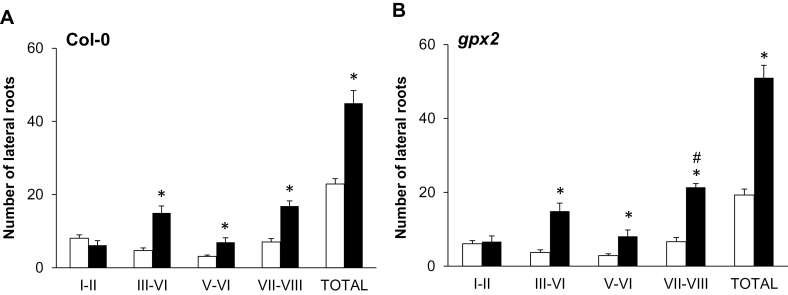
The effects of auxin on the stages of LRP development in the *gpx2* mutant relative to the wild type. The different stages of lateral root development were measured in seedlings of the *gpx2* mutant line (B) and the wild type (A) grown in either the absence (white columns) or the presence (black columns) of auxin (1 μM NAA). Significant differences (*P*<0.05) are indicated as follows: asterisks (*) denote differences between treatments in the same genotype, while hashes (#) denote differences between the mutant and wild type as a result of auxin treatment.

The initiation and early development of lateral root is regulated by auxin. The activation of the lateral root primordium (LRP), which occurs in a well-defined spatial order, was compared in the mutant and wild-type lines. For simplicity, the early steps of LRP development were grouped into different stages that are designated I (initiation) to VIII (emergence), based on the cell layers formed (Malamy and Benfey 1997). The detailed comparisons of the different stages of primordia development are shown here only for the *gpx2* mutants and Col-0 (Fig. 6). Auxin treatment significantly increased the number of primordia at developmental stages III–VIII in Col-0 and *gpx2* (as indicated by asterisks), relative to controls grown in the absence of auxin (Fig. 6). The number of primordia of stages I–II was not altered by auxin treatment in either genotype. GPX2 deficiency did not alter the auxin-dependent development of LRPs (Fig. 6).

### Effects of strigolactones and auxin on the abundance of AtGPX transcripts

**Fig. 7.**
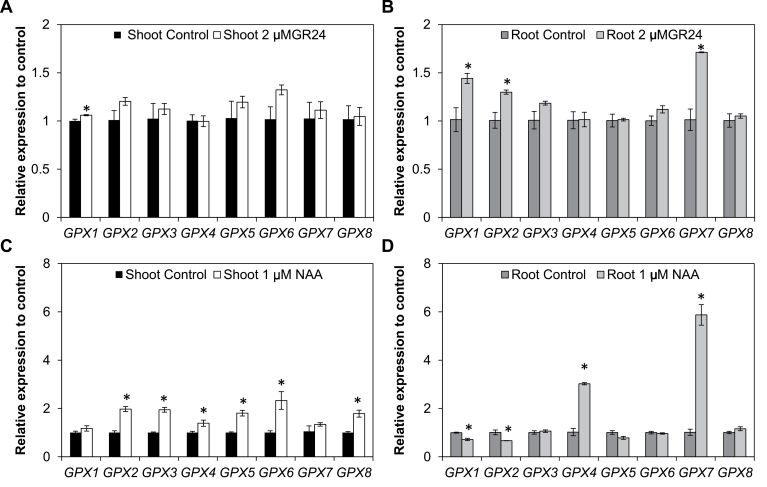
The effects of GR24 (2 μM; A, B) and NAA (1 μM; C, D) on the expression of the *GPX* genes in Col-0 roots and shoots. Black and white bars denote shoots before and after hormone treatment, respectively; whereas dark and light grey bars denote roots before and after hormone treatment, respectively. Data are the mean ±SE of three biological replicates; asterisks indicate significant differences, *P*<0.05, between treatment and control.

The effects of auxin (NAA) and SL (GR24) on the expression of *AtGPX* genes was characterized by comparing the effects of these hormones on the abundance of *AtGPX* transcripts in the roots and shoots of wild type seedlings. The addition of GR24 significantly increased *AtGPX1* mRNAs in shoots and roots relative to controls (Fig. 7A, B) and increased the levels of *AtGPX2* and *AtGPX7* mRNAs in roots (Fig. 7A, B). The addition of auxin led to a significant increase in the abundance of all the *AtGPX* mRNAs, except for those of *AtGPX1* and *AtGPX7* in shoots (Fig. 7C). In contrast, *AtGPX1* and *AtGPX2* transcript levels were lower in the roots of the auxin-treated seedlings. However, *AtGPX4* and *AtGPX7* mRNA levels were higher in the roots after auxin treatment (Fig. 7D).

## Discussion

GPXs are considered to play important roles in oxidative signalling and the metabolism of hydrogen peroxide, as well as the prevention of oxidant-induced PCD. The data presented here show that knockout mutations in each of the different GPX proteins had no adverse effects on leaf chlorophyll contents or rosette growth parameters. This finding would suggest that other components of the antioxidant system can compensate for the loss of GPX function in leaves in controlled growth conditions. Moreover, the *gpx7* mutants had a greater rosette diameter than the wild type under shorter photoperiod growth conditions and more leaves than the wild type under long photoperiod growth conditions. These data suggest that GPX7 protein has functions in the pathways that constrain shoot development. In this regard, it is interesting to note that the addition of auxin increased the abundance of all of the *GPX* mRNAs, except for those of *GPX7* and *GPX1* in the shoots (Fig. 7). AtGPX7 and AtGPX1 are known to fulfil important roles in chloroplasts, contributing to the cross-talk between high light stress and responses to pathogens (Chang *et al.*, 2009). A part of this cross-talk involves the control of growth and development under optimal and stress conditions. The data presented here suggest a role for AtGPX7 but not AtGPX1 in the pathways that control shoot growth.

The systematic characterization of the functions of the different GPX isoforms in the control of shoot and root phenotypes reported here demonstrates the importance of these enzymes in the control of root architecture, particularly lateral root development. There is little information in the literature concerning the functions of GPX in roots. The roots of the *gpx8* mutants are more sensitive to the oxidative stress induced by the herbicide methyl viologen (paraquat) than wild type roots (Gaber *et al.*, 2012). The data reported here suggest that the GPXs are important in the hormone-mediated regulation of lateral root development. GSH is involved in the interplay between auxin and SL signalling that controls this process (Marquez-Garcia *et al.*, 2013). For example, LRD was significantly decreased in GSH synthesis mutants (*cad2-1*, *pad2-1*, and *rax1-1*). Moreover, the application of GR24 increased the root GSH pool in a manner that was dependent on the MORE AXILLARY GROWTH (MAX) 2 signalling protein (Marquez-Garcia *et al.*, 2013).

Auxin regulates the development of the LRP (De Smet *et al.*, 2003). It triggers the first divisions of lateral root founder cells in the pericycle tissue of the primary root (Casimiro *et al.*, 2003) and later it accumulates at the tip of the LRP, where it promotes the degradation of Aux/IAA proteins (Péret *et al.*, 2009). Leaf-derived auxins are also important in the control of lateral root growth after emergence (Bhalerao *et al.*, 2002; Swarup *et al.*, 2008). The action of auxin is modulated by SLs, which influence auxin signalling pathways on multiple levels (Agusti *et al.*, 2011). SLs inhibit lateral root initiation (Kapulnik *et al.*, 2011) by blocking auxin transport (Bennett *et al.*, 2006; Crawford *et al.*, 2010; Balla *et al.*, 2011). It has previously been shown that NADP-linked thioredoxin and GSH systems are required for auxin transport and signalling (Bashandy *et al.*, 2010). Reduced thiols are also required for other processes that impact on root development. For example, the *gat1* mutant (gfp-arrested trafficking), which is defective in thioredoxin m3, accumulates high levels of callose leading to the occlusion of the plasmodesmata in the root meristem, resulting in arrested root development (Benitez-Alfonso *et al.*, 2009). The root phenotypes associated with the *gpx* mutants described here support the concept that redox processes involving glutathione and thioredoxins exert a strong influence on root development. GPXs may be required to mediate GSH and reduced thioredoxin functions in roots that impact on lateral root production or growth.

The data presented here demonstrate that *GPX1* and *GPX7* are important in the control of root architecture and lateral root development. Several plastid-localized enzymes that are important in the control of cellular redox homeostasis have also been shown to be required for root development. For example, the *miao* mutant is defective in the plastid-localized glutathione reductase (GR2) and consequently it has ~50% lower GR activity than the wild type and accumulates glutathione disulphide, GSSG (Yu *et al.*, 2013). The *miao* mutant shows impaired root growth with severe defects in root meristem maintenance, suggesting that a high plastid GSH:GSSG ratio is important for root development (Yu *et al.*, 2013). Conversely, severe GSH deficiency alone (without changes in the GSH:GSSG ratio) led to an arrest of the cell cycle at G_2_ in the root meristem with no apparent effect on root meristem maintenance (Schnaubelt *et al.*, 2014). Less severe GSH deficiency or depletion of only the cytosolic GSH pool resulted in decreased LRD (Schnaubelt *et al.*, 2013). A lack of reduced thioredoxin caused by mutation in the gene encoding NADPH-thioredoxin reductase (NTR) C led to decreased root elongation and lower rates of lateral root formation (Kirchsteiger *et al.*, 2012). The data presented here show that LRD values were higher in the *gpx1* and *gpx7* mutants than in the wild-type controls. Moreover, LRD values were more sensitive to auxin in the *gpx1* and *gpx7* mutants than in the wild type. These results strongly implicate the plastid-localized AtGPX1 and AtGPX7 enzymes in the auxin-dependent control of lateral root production. However, while *GPX7* transcripts were significantly increased in roots as a result of the application of either auxin or SL, *GPX1* transcripts were significantly more abundant following auxin treatment but significantly decreased in response to SL treatment. Hence, the hormone-dependent regulation of the AtGPX1 and AtGPX7 enzymes is different, at least at the level of gene expression. The *gpx1* and *gpx7* root phenotypes resemble the *gstu17* mutant, which exhibits altered auxin-dependent regulation of lateral root development (Jiang *et al.*, 2010). However, unlike the *gstu17* mutants that show a lower sensitivity to ABA, *gpx1* and *gpx7* mutants show responses to ABA similar to those of the wild type.

In contrast to *GPX1* transcripts, *GPX2* mRNA levels were decreased in roots following the application of auxin but increased following the application of SL. However, the roots of the *gpx2* mutants showed responses to SLs and to auxin very similar to those observed in the wild type, as did mutants deficient in other cytosolic GPX isoforms, such as *gpx4*. The *gpx3* mutants had a lower LRD than the wild type but they showed similar responses to the wild type in root architecture to the auxin, SL, and ABA treatments. Like rice plants deficient in GPX3, which showed an altered root structure with shorter roots than the wild type (Passaia *et al.*, 2013), the *gpx3* mutants studied here had shorter primary roots and fewer lateral roots than the wild type. The AtGPX3 protein has been shown to be important in ABA signalling under drought stress (Miao *et al.*, 2006). ABA suppresses the auxin response and is an inhibitor of lateral root development (De Smet *et al.*, 2003). The *abscisic acid insensitive* *4* ( *abi4*) mutant has an increased number of lateral roots relative to the wild type (Shkolnik-Inbar and Bar-Zvi, 2010). However, the responses of root architecture to ABA may vary between species because legume roots were shown to increase LRD in response to ABA (Liang and Harris, 2005). The data presented here show that roots of the *gpx3* mutants had similar responses to the wild type control to ABA, indicating that AtGPX3 is not required for ABA-dependent control of root architecture.

The data presented here show that the application of auxin increased the abundance of several GPX transcripts. Auxin is known to induce the expression of several glutathione *S*-transferases (GSTs) such as *AtGSTF2*. AtGSTF2 binds indole-3-acetic acid (IAA) and the auxin transport inhibitor 1-*N*-naphthylphthalamic acid (NPA), and so enhanced expression of GSTF2 might be linked to stress-mediated growth responses (Smith *et al.*, 2003). Mutants lacking another auxin-inducible GST, AtGSTU17, were less sensitive to auxin and had lower numbers of lateral roots in the presence of auxin (Jiang *et al.*, 2010). The *gstu17* mutants were also less sensitive to ABA-mediated inhibition of root development (Jiang *et al.*, 2010).

In conclusion, the data presented here demonstrate that the GPX proteins are important in the control of root architecture and that loss of any of the GPX isoforms exerts an influence on LRD. The plastid isoform AtGPX7 appears to be important in the control of shoot and root development by redox-dependent processes.

## Supplementary data

Supplementary data are available at *JXB* online.

Figure S1. The positions of T-DNA insertions (blue arrows) in the *gpx* mutants and the positions of the primers used to detect the presence of the insert (black arrows).

Table S1. The relative expression of *GPX* genes in different genotypes.

Table S2. Sequences of the primers used for the isolation of T-DNA insertion mutants (PCR; A and B) and for the gene expression analysis (qPCR; C).

Supplementary Data
