# Supplementary material for: The effects of redox controls mediated by glutathione peroxidases on root architecture in Arabidopsis thaliana
Source: J Exp Bot. 2014 Jan 27;65(5):1403–13. doi: 10.1093/jxb/ert486 (PMC3969529; doi:10.1093/jxb/ert486)
Supplement: Supplementary Data [file supp_65_5_1403__index.html]

The effects of redox controls mediated by glutathione peroxidases on root architecture in Arabidopsis thaliana — The effects of redox controls mediated by glutathione peroxidases on root architecture in Arabidopsis thaliana — Supplementary Data 

# The effects of redox controls mediated by glutathione peroxidases on root architecture in *Arabidopsis thaliana*

## Supplementary Data

Data files

**Files in this Data Supplement:**

- Supplementary Data - Supplementary Data
