# Supplementary material for: The effects of redox controls mediated by glutathione peroxidases on root architecture in Arabidopsis thaliana
Source: J Exp Bot. 2014 Jan 27;65(5):1403–13. doi: 10.1093/jxb/ert486 (PMC3969529; doi:10.1093/jxb/ert486)
Supplement: Supplementary Data [file supp_ert486_jexbot114413_file001.pdf]

Characterization of glutathione peroxidase mutants in *Arabidopsis thaliana*

Gisele Passaia, Guillaume Queval, Juan Bai, Marcia Margis-Pinheiro, and Christine H Foyer

Supplementary Data

Supplementary Table 1. The relative expression of *GPX* genes in different genotypes.

genotypes		Relative expression of *GPX* genes to *ACTIN2*							
		GPX1	*GPX2*	*GPX3*	*GPX4*	*GPX5*	*GPX6*	*GPX7*	*GPX8*
Shoot	Col0	0.583897	0.526283	0.038684	0.000586	0.047276	0.193305	0.028632	0.041958
	gpx1	0.051316	0.360232	0.030694	0.000426	0.045068	0.147439	0.024915	0.038181
	gpx2	0.662121	0.020765	0.02928	0.000393	0.038959	0.131308	0.036973	0.034295
	gpx3-2	0.643795	0.530478	1.13E-05	0.000582	0.060857	0.274692	0.02901	0.050836
	gpx4	0.906212	0.46194	0.036439	1.21E-06	0.055947	0.140097	0.068834	0.044079
	gpx6	0.666381	0.331125	0.030948	0.00038	0.041918	0.011768	0.028127	0.036447
	gpx7	0.673962	0.304144	0.02973	0.000348	0.05202	0.112239	0.004966	0.034872
	gpx8	0.61379	0.347808	0.033322	0.00035	0.046591	0.15147	0.030823	9.51E-05
Root	Col0	0.019749	0.563065	0.033667	0.000924	0.036026	0.457796	0.000226	0.068028
	gpx1	0.004984	0.388765	0.031013	0.000962	0.042936	0.372461	0.000128	0.065144
	gpx2	0.020583	0.009165	0.029497	0.00076	0.035361	0.353588	0.000115	0.06047
	gpx3-2	0.020031	0.627428	2.8E-06	0.000773	0.038166	0.470731	0.000118	0.064907
	gpx4	0.021439	0.536	0.034082	3.66E-07	0.047168	0.373592	0.000222	0.076151
	gpx6	0.021585	0.358378	0.029828	0.000833	0.035196	0.033799	9.59E-05	0.061661
	gpx7	0.020293	0.334922	0.028525	0.000718	0.038091	0.291132	0.000805	0.05347
	gpx8	0.019682	0.414514	0.031773	0.000767	0.0349	0.410112	8.1E-05	6.17E-05

Supplementary Table 2. Sequences of the primers used for the isolation of T-DNA insertion mutants (PCR; A and B) and for the gene expression analysis (qPCR; C).

A

Donor stock	Mutant	Forward	Reverse
SALK_027373	*gpx1*	5’-TGAAAATTCCTCGTGTTTTGG-3’	5’-TTTTCAAGTGTTCAAAGCGAAG-3’
SALK_082445	*gpx2*	5’-TTGTTTGCCACACATTGATTG-3’	5’-TTTTACCATTTGGAAGCAACG-3’
SAIL_278_E06	*gpx3-1*	5’-TTCAGTCGAAGAAGCAAACG-3’	5’-AACAGTTGCCAAACTCTTAGGG-3’
SALK_071176	*gpx3-2*	5’-TTGACTATAAGAAGCACTTTCCCG-3’	5’-GCATGGTTTGACGATTTTGTGA-3’
SALK_139870	*gpx4*	5’-GCAAGCTCATCTCCTTTGTTG-3’	5’-AAAAATGCCTGTCATGCATG-3’
WISCDSLOX_321_H10	*gpx6*	5’-CCATTGAGAAGCAACGTTCAC-3’	5’-TGGCCTCCTTTCTTTCTTTTG-3’
SALK_023283	*gpx7*	5’-AAGGGCTGCAGCAGAGAAG-3’	5’-TTGAACCGATCGAAAATTCC-3’
SALK_127691	*gpx8*	5’-CAGTGGGATGACAAACTCAAAC-3’	5’-CAGAACCACCCCACTTTCAG-3’

B

Collection	Mutant	T-DNA left border
SALK	*gpx1, gpx2, gpx3-2,gpx4, gpx7, gpx8*	5’-TGGACCGCTTGCTGCAACTCTC-3’
SAIL	*gpx3-1*	5’-AAATGGATAAATAGCCTTGCTTCC-3’
WISC	*gpx6*	5’-AACGTCCGCAATGTGTTATTAAGTTGTC-3’

C

AGI Code	Gene	Forward	Reverse
At2g25080	*GPX1*	5’-GTCTCCGGTAACCAAAAATG-3’	5’-GACGAGAAAGGTTGCTGAGG-3’
At2g31570	*GPX2*	5’-AAACTGCGTTGGGACAGG-3’	5’-CCCATGAAAAGACATCGAATAC-3’
At2g43350	*GPX3*	5’-GGGTCAATCAGCGAGCTAC-3’	5’-CGATGGCGAAGAAGGGTATC-3’
At2g48150	*GPX4*	5’-CCCACCTTTCTGGGTTCC-3’	5’-AACCATCGTGCCATAACG-3’
At3g63080	*GPX5*	5’-CAAAACGCTGCACCAGTC-3’	5’-GACCATCTTTGCCGACCAAG-3’
At4g11600	*GPX6*	5’-GATGTTAACGGTGACAAAGCTG-3’	5’-TTGGTGCGAAACGATCG-3’
At4g31870	*GPX7*	5’-TCGGCCCATCATTGAGATTC-3’	5’-CTGCAGCCCTTGCATAGAC-3’
At1g63460	*GPX8*	5’-GAAAGGCAAATGGGGAATC-3’	5’-CAGCTTGACCGTTTTTGTC-3’
At3g18780	*ACTIN2*	5’CTGTACGGTAACATTGTGCTCAG-3’	5’-CCGATCCAGACACTGTACTTCC-3’

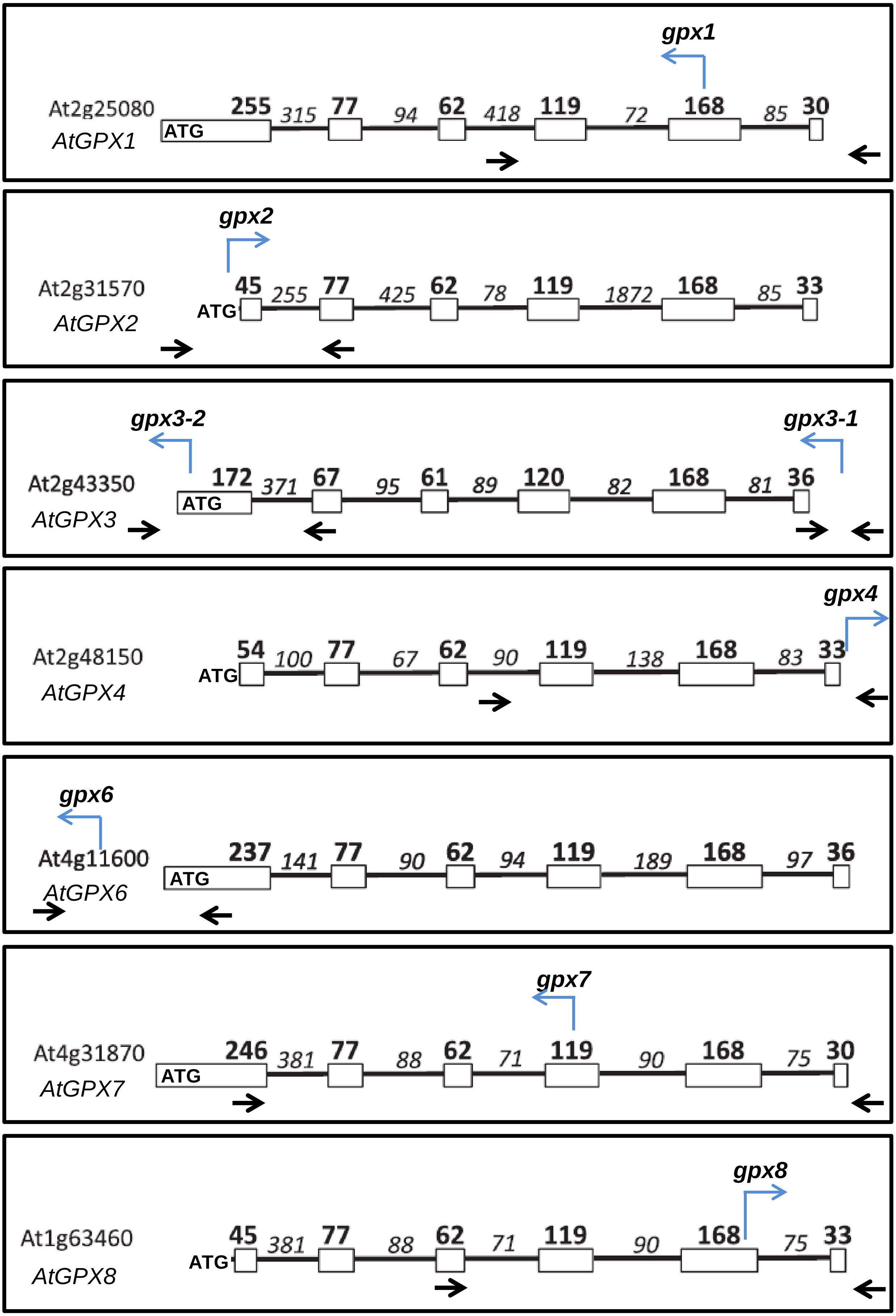

Supplementary Figure 1. The positions of T-DNA insertions (blue arrows) in the *gpx* mutants and the positions of the primers used to detect the presence of the insert (black arrows).
